# Plasma Drug Values of DOACs in Patients Presenting with Gastrointestinal Bleeding: A Prospective Observational Study

**DOI:** 10.3390/medicina59081466

**Published:** 2023-08-16

**Authors:** Dorotea Bozic, Damir Alicic, Dinko Martinovic, Ivan Zaja, Josipa Bilandzic-Ivisic, Rosana Sodan, Branka Kresic, Andre Bratanic, Zeljko Puljiz, Zarko Ardalic, Josko Bozic

**Affiliations:** 1 Department of Gastroenterology, Clinic for Internal Medicine, University Hospital of Split, Spinciceva 1, 21000 Split, Croatia; 2 Department of Maxillofacial Surgery, University Hospital of Split, Spinciceva 1, 21000 Split, Croatia; 3 Department of Gastroenterology, General Hospital of Sibenik-Knin County, Stjepana Radica 83, 22000 Sibenik, Croatia; 4 Department of Medical Laboratory Diagnostics, University Hospital of Split, Spinciceva 1, 21000 Split, Croatia; 5 Department of Internal medicine, University of Split School of Medicine, Soltanska 2, 21000 Split, Croatia; 6 Department of Pathophysiology, University of Split School of Medicine, Soltanska 2, 21000 Split, Croatia; josko.bozic@mefst.hr

**Keywords:** DOAC, NOAC, anticoagulant therapy, gastrointestinal bleeding, drug concentration

## Abstract

*Background and Objectives*: Anticoagulants are a well-known risk factor for gastrointestinal bleeding (GIB). In recent years, direct oral anticoagulants (DOACs) have taken a leading role in the treatment and prevention of thromboembolic incidents. The aim of this study was to investigate the prevalence of DOAC-treated patients with GIB whose plasma drug concentrations exceeded the cut-off values reported in the literature and to evaluate their clinical characteristics. *Materials and Methods*: Patients who were admitted to the Intensive Care Unit in the period 2/2020–3/2022 due to GIB were prospectively included in the study and classified into three groups according to the prescribed type of DOAC (apixaban, rivaroxaban, and dabigatran). For all participants, it was determined if the measured plasma drug levels exceeded the maximum serum concentration (C_max)_ or trough serum concentration (C_trough_) obtained from the available data. A comparison of clinical parameters between the patients with and without excess drug values was performed. Results: There were 90 patients (54.4% men) included in the study, of whom 27 were treated with dabigatran, 24 with apixaban, and 39 with rivaroxaban. According to C_max_, there were 34 (37.8%), and according to C_trough_, there were 28 (31.1%) patients with excess plasma drug values. A statistically significant difference regarding excess plasma drug values was demonstrated between DOACs according to both C_max_ (*p* = 0.048) and C_trough_ (*p* < 0.001), with the highest rate in the group treated with dabigatran (55.6% for C_max_ and 59.3% for C_trough_). Multivariate logistic regression showed that age (OR 1.177, *p* = 0.049) is a significant positive and glomerular filtration rate (OR 0.909, *p* = 0.016) is a negative predictive factor for excess plasma drug values. A total of six (6.7%) patients had fatal outcomes. *Conclusions*: Plasma drug concentrations exceed cut-off values reported in the literature in more than one-third of patients with GIB taking DOAC, with the highest rate in the dabigatran group. Clinicians should be more judicious when prescribing dabigatran to the elderly and patients with renal failure. In these patients, dose adjustment, plasma drug monitoring, or substitution with other, more appropriate DOACs should be considered.

## 1. Introduction

Oral anticoagulants are administered to a substantial number of patients globally, primarily for the purposes of stroke prevention in atrial fibrillation (AF) but also as a prophylactic or treatment method in venous thromboembolism (VTE) [1,2,3].

After the dominant use of vitamin K antagonists (VKA), direct oral anticoagulants (DOACs), which can act as factor IIa or Xa inhibitors, including dabigatran, apixaban, rivaroxaban, edoxaban, and betrixaban, have been introduced into clinical practice. In addition to their rapid onset of action, wide therapeutic window, lower predisposition for drug and food interactions, and no need for therapeutic monitoring, they also demonstrate high efficacy and a favorable safety profile, all of which have contributed to their increased global use [4,5].

However, concerns have been raised in regard to their application in the clinical environment as they carry a risk of bleeding, which is the most common complication of anticoagulant therapy. In addition to intracranial hemorrhage, which often leads to permanent disability or even death, gastrointestinal bleeding (GIB) has been the focus of attention due to its high prevalence and severe economic burden worldwide [3]. A large meta-analysis that included 43 randomized controlled trials and 41 real-world studies confirmed no significant difference in the risk of major GIB between the patients treated with DOACs compared to the ones receiving conventional anticoagulant therapy [3]. According to studies, the overall incidence of major GIB ranges between 0.5–1.6 and 0.8–1.9 events per 100 patient-years among patients treated for VTE and AF, respectively [6]. In comparison to the VKAs, which demand strict therapeutic monitoring and frequent dose adjustments, patients with DOACs are treated with fixed-dose regimens, and the measurement of drug plasma levels is not incorporated into clinical practice. 

Since it has not yet been determined whether the bleeding episodes are caused by elevated plasma drug values, we aimed to determine plasma drug concentrations in patients hospitalized for acute GIB. The primary endpoint was to investigate the rate of patients treated with one of the DOACs (rivaroxaban, apixaban, or dabigatran) whose plasma drug concentrations exceeded cut-off values reported in the literature. The secondary endpoint was to analyze patients’ clinical and endoscopic characteristics, treatment features, and outcomes, as well as their interrelation with plasma drug concentrations. 

## 2. Materials and Methods

Adult patients of both genders who were treated with DOACs (dabigatran, apixaban, rivaroxaban) and admitted to the Intensive Care Unit (ICU) of the Department of Gastroenterology at the University Hospital of Split due to upper or lower GIB, in a period between February 2020 to March 2022, were prospectively included in the study after signing the informed consent. 

Patients with severe anemia without manifest bleeding, suspected GIB that was excluded upon admission, and patients with iatrogenic bleeding (e.g., post polypectomy bleeding) were not included in the study. 

Patients’ hospital records were searched for age, sex, type of DOAC (apixaban, rivaroxaban, or dabigatran), DOAC dosing, indication for therapy (AF, deep venous thrombosis (DVT), pulmonary embolism), time elapsed from the last intake of drug to the blood coagulation sampling (expressed in hours), bleeding localization (esophagus, stomach, duodenum, small intestine, and large intestine) and the source of bleeding (Mallory Weiss syndrome, varices, ulcer, erosions, angiodysplasia, polyp, neoplasm, diverticula, colitis, or hemorrhoids), information about previous GI disorders (peptic ulcer, gastritis/duodenitis, neoplasm, inflammatory bowel disease, diverticula, liver cirrhosis, hemorrhoids, or pancreatitis), information about comedications (nonsteroidal anti-inflammatory drugs (NSAIDs), antiaggregant, antihypertensives, proton pump inhibitors (PPI), or selective serotonin reuptake inhibitors (SSRI)), days of hospitalization, number of received red blood cell (RBC) or fresh frozen plasma (FFP) doses, and information on receiving prothrombin complex (PCC) or dabigatran antidote (idarucizumab).

The following laboratory findings were documented: white blood cell count (WBC), RBC count, platelets (PLT), hemoglobin (Hb), prothrombin time (PT), international normalized ratio (INR), activated partial thromboplastin time (aPTT), fibrinogen, CRP, creatinine (Cr), Chronic Kidney Disease Epidemiology Collaboration glomerular filtration rate (CKD-EPI GFR), albumin, and bilirubin.

Charlson Comorbidity Index (predicts 10-year survival in patients with multiple comorbidities), HAS-BLED score (assesses the 1-year risk for major bleeding in patients taking anticoagulants for AF), CHA_2_DS_2_-VASc score (calculates stroke risk for patients with atrial fibrillation), and the Rockall Score (calculates the severity of bleeding from the gastrointestinal tract) were also determined. Additionally, we evaluated the following: type of endoscopic hemostasis (mechanical, pharmacological, or thermal), episodes of rebleeding, and the need for surgical treatment. The outcome was defined as survival or death. 

For each patient, one blood sample was taken immediately upon admission, prior to any transfusion treatment, and the period from the last ingestion of the drug (in hours) was recorded. The sample was sent to the Central Laboratory for the measurement of the plasma DOAC level, and centrifugation (1500× *g*, 15 min) was performed within one hour after blood sampling. Afterwards, samples were aliquoted and frozen immediately at −80 °C. They were thawed and heated to 37 °C for 5 min before testing. All samples were analyzed within one week after sampling. All tests were performed on a Atellica COAG 360 System coagulation analyzer (Siemens Healthineers, Erlangen, Germany). Dabigatran concentrations were measured using Innovance DTI assay (Siemens Healthineers, Erlangen, Germany) calibrated with Dabigatran Standards (Siemens Healthineers, Erlangen, Germany). Rivaroxaban and apixaban were measured using anti-Xa Innovance Heparin assay (Siemens Healthineers, Erlangen, Germany) calibrated with BIOPHEN™ Rivaroxaban Calibrator and BIOPHEN™ Apixaban Calibrator (Hyphen BioMed, Neuville-sur-Oise, France), respectively. 

We found data in the literature on the expected maximum serum concentration (C_max_) and trough serum concentration (C_trough_) for each drug (depending on the dose and indication), and accordingly defined excess plasma drug values in two ways: if they exceeded C_max_ or the C_trough_ level (Table 1) [7,8].

Patients with excess C_max_ values were defined if the level of the drug in the plasma was above the C_max_ value reported in the literature [7,8].

C_trough_ level is the expected concentration of the drug in the plasma detected immediately before the next dose administration. Patients with excess C_trough_ values were defined only if the plasma drug concentration exceeded the C_trough_ level reported in the literature 12 h after the last drug ingestion for apixaban and dabigatran (taken BID), and after 24 h for rivaroxaban (taken QD). If the time from the last drug ingestion was under 12 h (for apixaban and dabigatran), or under 24 h for rivaroxaban, the C_trough_ was defined as N.A. In the same manner, we labeled the patients who had the last drug ingestion substantially far from the C_trough_ time.

All procedures were carried out in accordance with the ethical standards of the institutional and national research committee and with the 1964 Helsinki Declaration and its later amendments or comparable ethical standards. All patients included in the study gave their informed consent. Ethical approval was given by the Ethics Committee of the University Hospital Split (No 500-03/20-01/09).

All statistical analyses were conducted using the computer software MedCalc (MedCalc Software, Ostend, Belgium, version 20.114). Categorical qualitative variables are presented as whole numbers (percentage) while continuous quantitative variables are presented as mean ± standard deviation and non-continuous quantitative variables are presented as median (interquartile range). The normality of distribution was estimated using the Kolmogorov-Smirnov test. Qualitative variables were compared between groups using the chi-square test. Parametric quantitative variables were compared between two groups using the student’s t-test for independent variables while non-parametric variables were compared using the Mann–Whitney U test. Moreover, correlations between parametric variables were calculated using Pearson’s correlation, while the correlations between non-parametric variables were calculated using Spearman’s correlation. Furthermore, independent predictors for intoxication with DOACs were estimated with multivariable logistic regression, with OR (odds ratio), 95% CI (95% confidence interval), and *p*-value reported. The multivariable logistic regression analysis was performed using the stepwise approach and the included variables were those of clinical significance according to the literature. Prior to the inclusion, the variables were tested for collinearity. Additionally, multiple linear regression analysis with a forward algorithm was conducted to determine significant independent predictors for the hospitalization duration. From these analyses, we have reported the *p*-values with unstandardized β-coefficients, standard error, and t-values. The level of statistical significance was set at *p*-value < 0.05.

## 3. Results

### 3.1. General Characteristics

There were 90 patients included in the study, out of whom 27 were treated with dabigatran, 24 with apixaban, and 39 with rivaroxaban at the time of admission. Among them, 49 (54.4%) were male and 41 (46.6%) were female patients with a high mean age of 79 ± 8 years. Most patients were administered DOAC for AF (67.8%). 

Regarding previous GI disorders, we found the highest prevalence of peptic ulcer disease or gastritis/duodenitis was present in 14 patients, 9 of whom were previously admitted for upper GIB. Regarding the history of gastrointestinal and hepatobiliary malignancies, one patient was treated for hepatocellular cancer, one for gastric cancer, and two for colorectal cancer. Diverticular disease was previously diagnosed in five, and colonic polyps in six patients. Upon admission, seven patients had elevated bilirubin levels, and two were previously treated for liver cirrhosis (Table 2). Regarding the CCI, which was used to categorize the severity of comorbidities, only 2 patients were classified in the mild grade (CCI 1–2), 23 in the moderate grade (CCI 3–4), and the majority (65 patients) in the severe grade (CCI ≥5). The median CCI was 5.5 (4.0–7.0), correlating with the severe comorbidity risk. One of the major contributors to the high CCI was renal failure since 56% of patients had CKD-EPI GFR corresponding with the chronic kidney disease grade 3–5 at admission. The median HAS-BLED score was 3, indicating a high risk for major bleeding. 

A small percentage of patients were treated with comedications that additionally aggravate the bleeding risk, such as NSAIDs (12.2%), SSRI (4.4%), and antiplatelets (7.8%). Only one-quarter of patients were treated with PPI upon admission (Table 2). We found a significantly higher percentage of patients with excess C_max_ values in the group taking NSAIDs (*p* = 0.017), while other medications showed no impact on the presence of elevated plasma drug values. Demographic, clinical, and laboratory values are systematically presented in Table 2.

### 3.2. Endoscopic Characteristics and Treatment

In most cases (39, 43.3%), the location of GIB was not specified. In patients where the location was defined, the most common source of bleeding was the stomach (22.2%), followed by the colon (18.9%), duodenum (10.0%), and, lastly, the esophagus (5.6%). In 40 (44.4%) patients, the source of bleeding could not be determined at the time of endoscopy. The most common sources of bleeding were peptic ulcers and erosions, which occurred in 30 patients overall (33.3%). 

In 22 patients (24.4%), hemostatic methods were needed for bleeding cessation. The mean duration of hospitalization was 7.5 (5.0–11.0) days. The mean number of RBC transfusions was 3.0 (2.0–5.0) doses, while the mean number of FFP transfusions was 2.0 (1.0–2.7) doses. Surgical treatment as the salvage method was indicated in 7 (7.8%) patients, and a total of 6 (6.7%) patients had fatal outcomes (Table 3).

### 3.3. DOAC Plasma Drug Values

**Scheme 1:**
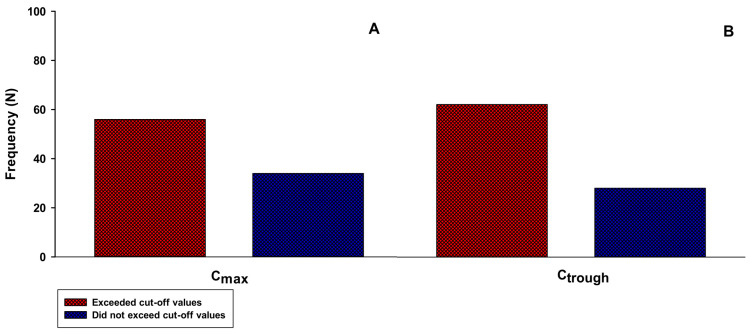
Distribution of patients in the study sample (N = 90) regarding their (**A**) C_max_ and (**B**) C_trough_ measurements.

According to the C_max_, there were 34 (37.8%), and according to the C_trough_, there were 28 (31.1%) patients with plasma drug concentrations exceeding the proposed cut-off values (Scheme 1). The median time since the last therapy intake was 17 (18–24) h.

Most of the patients with the excess plasma drug values were taking dabigatran (15 according to C_max_ and 16 according to C_trough_), and the majority of patients whose plasma drug concentrations did not exceed the proposed cut-off values reported in the literature were treated with rivaroxaban (29 according to C_max_ and 35 according to C_trough_).

Patients whose plasma drug concentrations exceeded the proposed cut-off values for C_trough_ had significantly longer PT (*p* = 0.033), lower CKD-EPI GFR (*p* < 0.001), and lower Rockall score (*p* = 0.036) values compared to those who did not have excess plasma drug concentrations (Table 4). Additionally, these patients had a significantly longer time since the last therapy intake (*p* = 0.023), longer APTT (*p* < 0.001) and higher creatinine values (*p* < 0.001) (Table 4).

There was no significant difference between patients regarding their C_max_ values and indications for DOAC (patients with excess plasma drug concentrations vs. non-excess: AF = 22 (64.7%) vs. 39 (69.6%); DVT = 3 (8.8%) vs. 4 (7.1%); PE = 2 (5.9%) vs. 3 (5.4%); other = 7 (20.6%) vs. 10 (17.9%); *p* = 0.969). Furthermore, there was also no significant difference between patients regarding their C_trough_ values and indications for DOAC (patients with excess plasma drug concentrations vs non-excess: AF = 19 (67.9%) vs. 42 (67.7%); DVT = 4 (14.3%) vs. 3 (4.8%); PE = 2 (7.1%) vs. 3 (4.8%); other = 3 (10.7%) vs. 14 (22.6%); *p* = 0.279).

Patients whose plasma drug concentrations exceeded the proposed C_max_ values had significantly shorter time since the last therapy intake (*p* = 0.007), a lower RBC count (*p* = 0.033), Hb concentration (*p* = 0.014), and CKD-EPI GFR (*p* < 0.001), as well as longer PT (*p* < 0.001) compared to the group of patients whose plasma drug concentrations did not exceed the proposed C_max_ values (Table 5). Additionally, these patients had significantly longer APTT (*p* = 0.022) and higher creatinine values (*p* = 0.011) and RBC transfusion doses (*p* = 0.025) (Table 5).

**Scheme 2:**
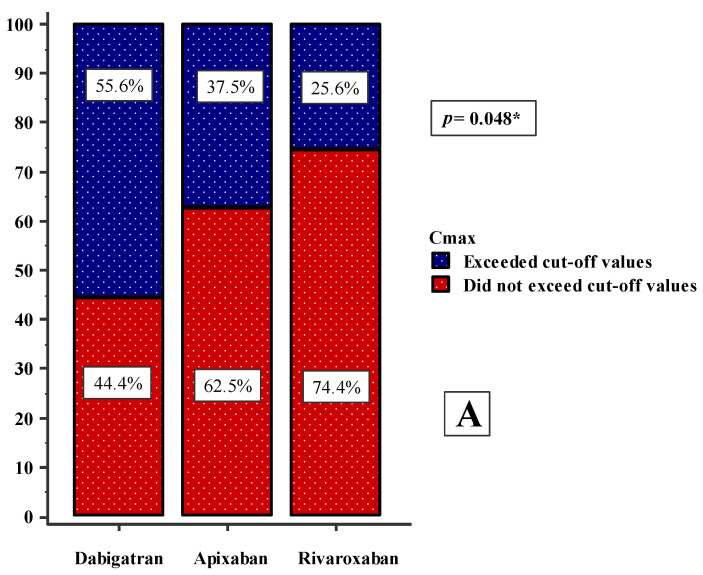

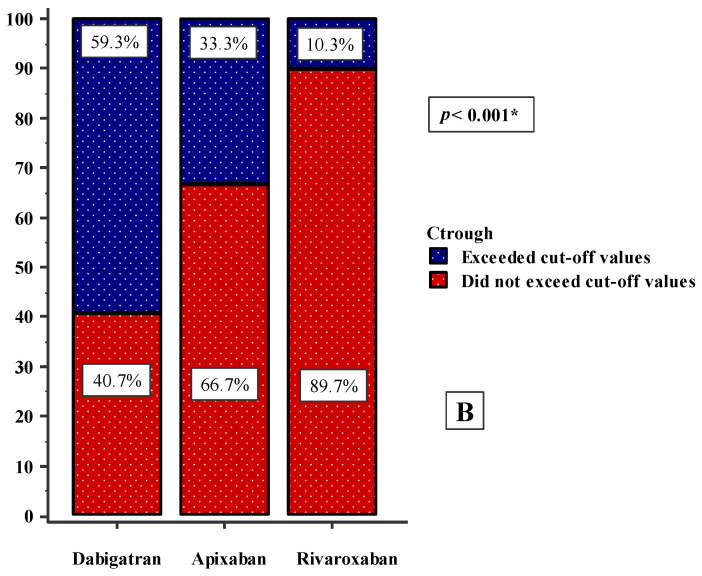
Comparison of the frequency (%) of patients with excess plasma drug concentrations between dabigatran (N = 27), apixaban (N = 24) and rivaroxaban (N = 39) according to the (**A**) C_max_ and (**B**) C_trough_ measurements. * chi-square test.

There was a statistically significant difference between DOACs in the rate of patients with excess plasma drug concentrations according to both C_max_ (*p* = 0.048) and C_trough_ (*p* < 0.001) (Scheme 2). In the group of patients treated with dabigatran, 55.6% had elevated drug levels according to the C_max_, while these rates in the apixaban and rivaroxaban groups were 37.5% and 25.6%, respectively (Scheme 2A). Likewise, in the group of patients treated with dabigatran, 59.3% of patients had excess plasma drug values, while this percentage was significantly lower in the groups of patients treated with apixaban (33.3%) or rivaroxaban (10.3%), according to the C_trough_ (Scheme 2B).

There was a significant positive correlation between the plasma levels of dabigatran and creatinine (r = 0.622; *p* < 0.001) values and a significant negative correlation with the CKD-EPI GFR (r = −0.640; *p* < 0.001). There were no significant correlations between plasma levels of apixaban or rivaroxaban with any of the clinical and laboratory parameters in the study sample. 

Multivariable logistic regression analysis showed that age (OR 1.117, 95% CI 0.999–1.387, *p* = 0.049) was a significant positive predictor, while the CKD-EPI GFR (OR 0.909, 95% CI 0.841–0.982, *p* = 0.016) was a significant negative predictor for the DOAC plasma concentrations exceeding the proposed C_trough_ cut-off values (Table 6). 

Multiple linear regression analysis showed that WBC count (β ± SE, 0.203 ± 0.088, *p* = 0.024), APTT (0.058 ± 0.030, *p* = 0.049), and the CCI (0.726 ± 0.278, *p* = 0.011) were significant positive predictors of hospitalization duration (Table 7). 

## 4. Discussion

We revealed that DOAC plasma concentrations exceed the cut-off values proposed in the literature in more than one-third of patients admitted to the Intensive Care Unit due to GIB. The highest proportion of patients with excess plasma drug values was found in the group of patients treated with dabigatran. 

There is a well-known paradigm that considers laboratory monitoring of DOAC anticoagulant activity unnecessary in clinical practice since these drugs have stable pharmacokinetic and pharmacodynamic profiles and are therefore administered in fixed doses [7]. However, experts in the field agree that their anticoagulant activity control is needed in certain urgent clinical settings, such as perioperatively, before invasive interventions, in bleeding scenarios, or in the occurrence of thromboembolic events despite regular treatment, but also in patients with extreme body weight or with end-stage renal failure [8,9]. Classical laboratory parameters such as PV or APTT are considered inappropriate in the determination of DOAC anticoagulant activity, although patients in our cohort that showed excess C_max_ and C_trough_ values had significantly longer PT (*p* < 0.001 and *p* = 0.033) and APTT (*p* = 0.022 and *p* < 0.001), respectively, meaning these parameters might be of clinical importance. 

The most precise and accurate method to ascertain their anticoagulant activity is the quantification of DOAC plasma levels, but this approach is considered expensive, requires laboratory expertise and local validation, and is therefore not widely available, especially in urgent settings [9,10].

Several authors investigated whether a single plasma drug level measurement could reliably identify patients with high drug levels. De Vries et al. demonstrated that patients who have high trough and peak levels of apixaban on a single occasion will usually retain high drug concentrations when the assay is repeated in 2 months. That being the case, the single finding of a high apixaban level might lead physicians to consider treating the patient with a lower apixaban dose [11]. Contrary to this, Shyamkumar K. et al. do not support this approach in patients treated with rivaroxaban, since only 26.7% and 13.3% of patients with a peak or trough level in the upper quintile retained the high drug level [12].

Although DOAC concentration level monitoring and dose adjustment are currently not recommended due to the lack of reliable tests and clinical evidence, certain patients would undoubtedly benefit whom this approach. In 2015, a meeting to discuss this issue was held at the American College of Cardiology Heart House with the conclusion that in the presence of a reliable licensed test, ‘monitor and adjust’ paradigm may be useful in patients presenting with DOAC overdose, major bleeding, undergoing thrombolysis, urgent surgery or invasive procedures; but also in patients with recurrent thromboembolic events, elderly (>75 years), patients with renal failure, extreme body weight (<50 kg or >110 kg), or with potential drug-drug interactions [13].

The results from our study support this thesis due to the unexpectedly high rate of patients with excess plasma drug concentrations (37.8% according to C_max_ and 31.1% according to C_trough_). The awareness of this issue is underestimated since there have been no published clinical studies on this matter. It is also important to highlight the statistically significant difference between the DOACs (*p* = 0.048 for C_max_ and *p* < 0.001 for C_trough_), with the highest rate of excess plasma drug concentrations being detected in the dabigatran group (55.6% and 59.3%), followed by apixaban (37.5% and 33.3%) and, finally, rivaroxaban (25.6% and 10.3%), according to the C_max_ and C_trough_, respectively. Patients with excess plasma drug values had significantly lower RBC (*p* = 0.033) and Hb concentrations (*p* = 0.014), as well as a greater number of RBC transfusions (*p* = 0.025), indicating the impact of the drug level on the severity or duration of bleeding. 

A particularly important group are patients with renal failure that are at high risk of drug accumulation and intoxication, especially in the setting of dabigatran treatment. This well-known paradigm is also proven in our study since we found that patients with excess C_max_ and C_trough_ values had significantly lower CKD-EPI GFR than patients without excess drug levels (*p* < 0.001). GFR was also a significant negative predictor for excess C_trough_ values in the multivariable logistic regression analyses. However, a significant negative correlation with the GFR (r = −0.640; *p* < 0.001) was found only with the plasma levels of dabigatran. Accordingly, regular monitoring of renal function, especially during the episodes of hypovolemia or acute infection in the elderly, is of the utmost importance. 

Apart from renal function, the other significant factor that is unfortunately too often neglected in clinical practice is the patient’s age. A remarkable proportion (75%) of patients in our cohort were older than 75 years, with a mean age of 78.8 ± 8.3 years. Multivariable logistic regression analysis showed that age (OR 1.117, 95% CI 0.999–1.387, *p* = 0.049) was the only significant positive predictor for excess C_trough_ values. Since a higher age carries an increased embolic risk, the CHA2DS2-VASc score was designed in such a way that all patients ≥ 75 years of age are recommended to take anticoagulants irrespective of the presence of additional risk factors [14]. Contrary to this, the HAS-BLED score indicates that the bleeding risk carries only 1 point for those aged ≥ 65 years. Contrary to the dose reduction recommendations in the elderly for dabigatran and apixaban, which are based on large clinical trials that included 30–44% of patients ≥ 75 years of age, there are no age-related dose reductions in patients treated with rivaroxaban [14]. A nationwide cohort study that included 30,401 patients ≥ 75 years found sub-hazard ratios against warfarin for a major bleeding event of 0.96 for rivaroxaban, 0.75 for dabigatran, and 0.74 for apixaban [15]. It is important to emphasize that apart from the manufacturer-recommended dose reductions, off-label underdosing may be hazardous, according to a recently published meta-analysis that found no effect of inappropriate dosing on bleeding outcomes (HR = 1.04, 95% CI 0.90–1.19; *p* = 0.625) but an increased risk of all-cause mortality (HR = 1.28, 95% CI 1.10 to 1.49; *p* = 0.006) [16]. Taking into account the aforementioned data, we may conclude that the elderly is at a higher bleeding risk, which seems to be caused by the elevated plasma drug concentrations, presumably in combination with low GFR and possibly drug overdosing despite the manufacturer’s instructions. We advise implementation of drug concentration monitoring in this complex group, which evidently makes up the majority of hospitalized patients due to DOAC-related GIB. 

We also advise the higher utilization of the HAS-BLED score in clinical practice. Among 61 patients with AF, 52 of them had a high (≥3) HAS-BLED score, which indicated an elevated bleeding risk. The HAS-BLED score, which assesses the 1-year risk for major bleeding events in patients treated with oral anticoagulants, serves as an alert to clinicians in order to provide careful and regular monitoring of high-risk patients. Lip GYH et al. conducted a large retrospective cohort study that included a total of 381 054 patients with a high GIB risk, among which 74.7% had HAS-BLED ≥3, and found the highest risk for the bleeding event in patients treated with rivaroxaban (HR, 1.11; 95% CI, 1.05–1.16), in comparison to those treated with dabigatran and apixaban (HR, 0.78; 95% CI, 0.70–0.86 and HR 0.59; 95% CI, 0.56–0.63, respectively) [17]. We found a significant rate of patients (85%) with a high HAS-BLED score in the subgroup of patients with AF, which is an additional confirmation of the need for an individualized approach, adapted DOAC choice, and regular monitoring of these patients.

Regarding their efficacy and safety in relation to VKAs, several clinical trials demonstrated DOACs having non-inferior or even superior efficacy and similar safety in comparison with warfarin [8]. A large meta-analysis that included 42,411 participants treated with DOAC and 29,272 participants treated with warfarin for AF who were included in four large trials (RE-LY, ROCKET AF, ARISTOTLE, and ENGAGE AF-TIMI 48) found that DOACs significantly reduced stroke or systemic embolic events by 19% compared with warfarin. They also significantly reduced overall mortality and intracranial hemorrhage but increased the GIB rate (RR 1.25, *p* = 0.04). However, the GIB rate was similar between low-dose DOACs and warfarin [18,19,20,21]. Furthermore, dabigatran plasma concentrations were obtained from 9183 patients in the RELY trial and showed dependence on age, gender, weight, and renal function. The patients who had a major bleeding event during the trial had higher trough and post-dose concentrations than subjects without bleeding events [22]. We may conclude that the equal safety of DOACs compared to standard treatment does not refer to the element of GIB.

Contrary to the abundant studies comparing efficacy and bleeding risk between patients taking DOACs and warfarin, studies comparing GIB rates among different DOACs are limited [23]. A nationwide population-based cohort study on 5868 patients taking DOACs in Iceland found that rivaroxaban is associated with higher GIB rates than apixaban and dabigatran, regardless of treatment indication [23]. Abraham NS et al. investigated the GIB safety profile between DOACs and also found that GIB occurred more frequently in patients given rivaroxaban than dabigatran (hazard ratio (HR) 1.20; 95% CI 1.00–1.45), while apixaban was associated with a lower risk of GIB than dabigatran (HR 0.39; 95% CI, 0.27–0.58; *p* < 0.001) or rivaroxaban (HR, 0.33; 95% CI, 0.22–0.49; *p* < 0.001) [24]. A Norwegian study on a cohort of 52,476 new users of DOACs for FA revealed the following hazard ratios (HRs) for GIB risk: 1.04 (95% CI 0.93–1.16) for dabigatran vs. rivaroxaban, 1.48 (95% CI 1.28–1.70) for dabigatran vs. apixaban, and 0.68 (95% CI 0.60–0.77) for apixaban vs. rivaroxaban [25]. Sherid M et al. carried out a head-to-head comparative study between dabigatran and rivaroxaban that included 374 patients (147 rivaroxaban vs. 227 dabigatran) and found no difference in the GIB risk between the groups (*p* = 0.8215) [26].

These studies indicate that apixaban has the most favorable GIB safety profile, while the GIB risk between dabigatran and rivaroxaban seems similar if not higher in rivaroxaban users. In our cohort of 90 patients, the highest proportion was treated with rivaroxaban (43%), while simultaneously these patients had the lowest rates (10–25%) of excess plasma drug concentrations, assuming the drug level would not likely be the cause of bleeding in these patients. 

According to Cheung KS and Leung WK, DOACs can cause GIB through several mechanisms, including their incomplete absorption with topical anticoagulant effect, a direct caustic effect due to dabigatran tartaric acid core, and the inhibition of mucosal healing [27].

In our cohort, the location of GIB was not specified in 43.3% of cases, and the source of the bleeding could not be determined in 44.4% of patients at the time of endoscopy. We assume the reason for such results was the termination of bleeding before the endoscopy procedure. Similarly, patients who bled from the small intestine were not detected during hospitalization since most of the video capsule endoscopies were performed in the post-hospitalization assessment. Although a high proportion of patients already had peptic ulcer disease or gastritis/duodenitis (42.4%) in their medical history, only 26.7% were taking PPI before admission. Consequently, the most common causes of bleeding were ulcers and erosions (33.3%). Similar to our study, where 37.8% of patients bled from the upper GI tract, other studies reported comparable rates of upper GIB (33.3–44.1%), mostly from peptic ulcers [28,29]. Co-treatment with PPI is recommended by a substantial number of authors due to the protection of the gastric mucosa, particularly in patients with a history of gastroduodenal ulcer disease [29,30,31]. A cohort study that included 164,290 patients initiated with DOAC for AF from 2011 until 2018 found that PPI use is associated with lower UGIB rates (incidence rate ratio (IRR): 0.75; 95% CI: 0.59–0.95), with the strongest impact on patients with a high HAS-BLED score (≥3), the elderly, and those undergoing concomitant antiplatelet therapy [31].

While Pannach S et al. reported a high percentage of lower GIB (42%), mostly caused by hemorrhoidal bleeding (33.3%), our rates regarding this pathology were significantly lower (18.9% for lower GIB and 2.2% for hemorrhoidal bleeding) [28]. Diamantopoulou G et al. also reported lower rates of hemorrhoidal bleeding (4.6%), with the highest proportion of patients bleeding from angiodysplasias and polyps/neoplasia (18.6% and 30.2%) [32].

Regarding the treatment methods in our cohort, 24.4% of patients were actively treated to achieve bleeding cessation, including mechanical (12.2%), thermal (2.2%), and pharmacologic hemostasis (10%). Surgical treatment was indicated in 7 (7.8%) patients. Among 43 patients admitted for lower GIB in the Greek cohort, 39.5% required endoscopic hemostasis and only 1 patient underwent surgical treatment [32]. The reported hospitalization duration (4.5 ± 3.6–6.9 ± 5.4 days) in other studies was somewhat shorter than in our cohort (8.2 ± 4.4 days) [19,27,29]. Multiple linear regression analysis found WBC count (β ± SE, 0.203 ± 0.088, *p* = 0.024), APTT (0.058 ± 0.030, *p* = 0.049), and the CCI (0.726 ± 0.278, *p* = 0.011) as significant positive predictors of hospitalization duration, indicating that it is usually prolonged due to concurrent infection or the presence of comorbidities. Patients generally required a low number of RBC transfusion (3.5 ± 2.2) doses, which coincides with other studies reporting lower transfusion requirements in patients with GIB treated with DOACs than in VKA or antiplatelet drug recipients [28,32].

Our cohort had a high mean age (78.8 ± 8.3) as well as a high CCI correlating with severe comorbidities. The ensuing mortality rate was 6.7%, similar to other studies (6.9% and 7%) [27,29]. In our previously published retrospective study of patients taking anticoagulant or antiplatelet therapy, a similar mortality rate (8.3%) was detected [33]. Contrary to this, Pannach C et al. demonstrated a lower mortality rate (1.7%) in their cohort of patients treated with DOACs compared to the groups treated with VKAs (5.6%) or antiplatelets (11.9%) [28].

According to ESGE guidelines on the treatment of upper GIB, DOAC should be immediately withheld and the patient should undergo endoscopic evaluation without delay. It is important to consider the time of the last DOAC intake since they are usually rapidly removed from the system owing to their short half-life, which is nevertheless the reason they are generally recognized as a safer treatment choice. In this context, renal function must be considered, particularly in patients treated with dabigatran [34]. ESGE also guides clinicians not to immediately interrupt DOAC treatment in cases of mild self-limiting lower GIB but to temporarily withhold it in cases of major lower GIB [35]. In cases of severe bleeding followed by hemodynamic instability, the use of a reversal agent or intravenous PCC should be considered [34,35].

Reversal agents include idarucizumab for dabigatran and andexanet alfa for rivaroxaban and apixaban [27,36]. The unfavorable side of these reversal agents is their exceptionally high price and the lack of availability in all centers. According to the Working Group on Perioperative Haemostasias, if the plasma drug level is ≤30 ng/mL, hemorrhage is unlikely to be caused only by a drug, and usually does not require anticoagulant reversal [10]. As in other similar studies, PCC and idarucizumab were not commonly used in our cohort, and andexanet alfa was not yet available during the study’s conduct [18,32]. Hemodialysis can be used as the salvage method in the event of dabigatran intoxication when the CrCl is <30 mL/min or in acute kidney injury, since it may remove 49–57% of dabigatran within 4 h [37]. No guidelines recommend the use of FFP, and according to the American Heart Association, there is no evidence that either FFP or PCC can control DOAC-related bleeding [34,35,37]. Anticoagulation should be restarted following the bleeding cessation, preferably within or soon after 7 days of the bleeding event [34,35].

The individualized treatment approach has taken a huge step forward in all fields of medicine, and it seems that the universal dosing regimen is lacking support in daily clinical practice. Certain authors agree about an evident need for a randomized controlled trial of fixed versus adjusted DOAC dosing regimen despite the probable high price and long duration of such a study, especially if we keep in mind that one-third of hospitalizations due to GIB episodes associated with exceeded plasma drug concentrations could be prevented [13].

## 5. Limitations 

There are currently no standardized data on the DOAC C_max_ and C_trough_ cut-off values. The analysis was performed using the cut-off values reported in the literature.

## 6. Conclusions

Our study demonstrates that DOAC plasma levels exceed the cut-off values reported in the literature in more than one-third of patients with GIB, with the highest rate in the dabigatran group. Therefore, clinicians should be more judicious when prescribing dabigatran to the elderly and patients with renal failure. In these patient groups, dose adjustment, plasma drug monitoring, or substitution with other, more appropriate DOACs should be considered. Additionally, measurement of plasma drug levels is recommended in cases of major bleeding to determine the impact of drug anticoagulant activity on the bleeding episode and to direct the choice of treatment, particularly regarding the need for antidote application.

## Figures and Tables

**Table 1:** Expected C_max_ and C_trough_ levels for each drug depending on the dose and indication [7,8].

Indication				
	AF	DVT/PE	AF	DVT/PE
**DABIGATRAN**	**150 mg bid**	**150 mg bid**	**110 mg bid**	
Cmax (ng/mL)	175	175	126	
Ctrough (ng/mL)	91	60	65	
**RIVAROXABAN**	**20 mg qd**	**20 mg qd**	**15 mg qd**	**10 mg qd**
C max (ng/mL)	249	270	229	101
C trough (ng/mL)	44	26	57	14
**APIXABAN**	**5 mg bid**	**5 mg bid**	**2.5 mg bid**	**2.5 mg bid**
C max (ng/mL)	171	132	123	67
C trough (ng/mL)	103	63	79	32

C_max_: maximum serum concentration, C_trough_: trough serum concentration; AF: atrial fibrillation; DVT: deep venous thrombosis; PE: pulmonary embolism; bid: two times a day; qd: one daily.

**Table 2:** Sociodemographic, clinical and laboratory parameters.

Parameter	Study Sample N = 90
Age (years)	78.8 ± 8.3
Male gender (N, %)	49 (54.4)
Indication (N, %)	
AF	61 (67.8)
DVT	7 (7.8)
PE	5 (5.6)
Other	17 (18.9)
DOAC type (N, %)	
Dabigatran	27 (30)
Apixaban	24 (26.7)
Rivaroxaban	39 (43.3)
Earlier GI disease (N, %)	
Peptic ulcer or gastritis/duodenitis	14 (15.5)
Neoplasm	4 (4.44)
IBD	0 (0)
Diverticula	5 (5.55)
Polyp	6 (6.66)
Liver cirrhosis	2 (2.2)
Hemorrhoidal disease None	2 (2.2) 57 (63.3)
CCI score	5.5 (4.0–7.0)
Rockall score	4.0 (3.0–5.0)
HAS-BLED ^†^ score	3.0 (3.0–4.0)
CHA_2_DS_2_-VASc ^†^ score	4.0 (4.0–5.0)
Laboratory parameters	
WBC (×10^9^/L)	9.6 (7.0–12.2)
RBC (×10^9^/L)	3.0 ± 0.8
Hb (g/L)	89.0 (67.0–115.0)
Platelets (×10^9^/L)	226.5 (175.0–288.0)
PT (ratio)	0.66 ± 0.24
INR (ratio)	1.2 (1.1–1.4)
APTT (sec)	27.1 (23.7–37.6)
Fibrinogen (g/L)	3.3 ± 0.8
hs-CRP (mg/L)	13.2 (3.4–39.0)
Creatinine (μmol/L)	104.5 (78.0–168.0)
CKD-EPI GFR (mL/min/1.73 m^2^)	55.3 (30.8–74.8)
Total bilirubin (μmol/L)	10.0 (6.0–15.3)
Albumin (g/L)	33.4 ± 7.5
NSAID (N, %)	
Yes	11 (12.2)
No	79 (87.8)
Antiplatelet drug (N, %)	
Yes	7 (7.8)
No	83 (92.2)
Antihypertensive drug (N, %)	
Yes	55 (61.1)
No	35 (61.1)
PPI (N, %)	
Yes	24 (26.7)
No	66 (73.3)
SSRI (N, %)	
Yes	4 (4.4)
No	86 (95.6)

All data are presented as whole number (percentage), mean ± standard deviation or median (interquartile range). DOAC—direct oral anticoagulants; GI—gastrointestinal; IBD—inflammatory bowel disease; CCI—Charlson Comorbidity Index; WBC: white blood cells; RBC: red blood cells; Hb: hemoglobin; PT—prothrombin time; INR—international normalized ratio; APTT—activated partial thromboplastin time; CRP—C reactive peptide; CKD-EPI—Chronic Kidney Disease Epidemiology Collaboration; GFR—glomerular filtration rate; NSAID—non-steroidal anti-inflammatory drugs; PPI—protein pump inhibitor; SSRI—selective serotonin reuptake inhibitor. ^†^ only patients with AF (N = 61).

**Table 3:** Endoscopic characteristics and treatment data.

Parameter	Study Sample N = 90
Cause of GIB (N, %)	
Not found	40 (44.4)
Mallory Weiss syndrome	0 (0)
Peptic ulcer	19 (21.1)
Erosions	11 (12.2)
Angiodysplasia	3 (3.3)
Polyp	2 (2.2)
Neoplasm	7 (7.8)
Diverticula	6 (6.7)
Colitis	0 (0)
Hemorrhoids	2 (2.2)
Forrest for ulcer disease (N, %)	
Ia	0 (0)
Ib	5 (26.3)
IIa	1 (5.3)
IIb	1 (5.3)
IIc	0 (0)
III	12 (63.2)
Mechanical hemostasis (N, %)	11 (12.2)
Thermal hemostasis (N, %)	2 (2.2)
Pharmacologic hemostasis (N, %)	9 (10)
Hospitalization duration (days)	8.2 ± 4.4
RBC transfusion (doses)	3.5 ± 2.2
FFP transfusion (doses)	2.4 ± 2.6
PCC (N, %)	
Yes	1 (1.2)
No	89 (98.9)
Idarucizumab (N, %)	
Yes	1 (1.2)
No	89 (98.9)
Rebleeding (N, %)	
Yes	1 (1.1)
No	89 (98.9)
Surgery (N, %)	
Yes	7 (7.8)
No	83 (92.2)
Outcome (N, %)	
Fatal	6 (6.7)
Non-fatal	84 (93.3)

All data are presented as a whole number (percentage) or median (interquartile range). FFP—fresh frozen plasma; PCC—prothrombin complex concentrate.

**Table 4:** Comparison of clinical parameters between the patients with excess vs. non-excess plasma drug concentrations according to the C_trough_ [7,8].

Parameter	Exceeded Cut-Off Values N = 28	Not Exceeded Cut-Off Values N = 62	*p* *
Age (years)	80.0 ± 7.0	78.3 ± 8.8	0.369 ^†^
Male gender (N, %)	14 (50.0)	35 (56.5)	0.733 *
Time since last therapy (hours)	21.0 (13.2–24.0)	14.5 (7.0–24.0)	0.023 *
WBC (×10^9^/L)	11.4 (7.1–13.5)	9.5 (7.0–11.4)	0.178 *
RBC (×10^9^/L)	2.9 ± 1.0	3.1 ± 0.7	0.373 ^†^
Hb (g/L)	91.5 (62.0–119.0)	86.0 (69.0–115.0)	0.934 *
Platelet (×10^9^/L)	248 (173–305)	220 (176–285)	0.507 *
PT (ratio)	0.58 ± 0.28	0.70 ± 0.21	0.033 ^†^
APTT (sec)	37.6 (25.3–52.0)	26.3 (23.1–30.3)	<0.001 *
Creatinine (μmol/L)	158.5 (113.0–191.5)	88.0 (72.0–128.0)	<0.001 *
CKD-EPI GFR (mL/min/1.73 m^2^)	31.2 (27.0–45.5)	65.0 (42.1–82.8)	<0.001 *
CCI (score)	5.0 (4.0–7.0)	6.0 (4.0–7.0)	0.599 *
Rockall (score)	4.0 (2.5–4.5)	4.0 (3.0–6.0)	0.036 *
Hospitalization duration (days)	8.5 (5.5–12.0)	7.0 (5.0–9.0)	0.288 *
RBC transfusion ^†^ (doses)	3.0 (2.0–5.0)	3.0 (2.0–5.0)	0.908 *
FFP transfusion (doses)	2.0 (1.2–3.0)	1.5 (1.0–2.0)	0.339 *

All data are presented as median (interquartile range). ^†^ student’s *t*-test for independent variables, * Mann–Whitney U test.

**Table 5:** Comparison of clinical parameters between the patients with excess vs. non-excess plasma drug concentrations according to the C_max_ [7,8].

Parameter	Exceeded Cut-Off Values N = 34	Not Exceeded Cut-Off Values N = 56	*p* *
Age (years)	78.7 ± 7.0	78.9 ± 9.1	0.892 ^†^
Male gender	16 (47.1)	33 (58.9)	0.380 *
Time since last therapy (hours)	11.0 (7.0–20.0)	20.5 (12.0–24.0)	0.007 *
WBC (×10^9^/L)	9.1 (6.9–12.6)	9.9 (7.3–12.1)	0.644 *
RBC (×10^9^/L)	2.8 ± 0.8	3.1 ± 0.7	0.033 ^†^
Hb (g/L)	69.5 (58.0–111.0)	97.5 (78.0–117.5)	0.014 *
Platelet (×10^9^/L)	253 (173–309)	220 (182–276)	0.340 *
PT (ratio)	0.53 ± 0.25	0.74 ± 0.19	<0.001 ^†^
APTT (sec)	29.6 (24.4–48.2)	26.3 (23.1–30.4)	0.022 *
Creatinine (μmol/L)	142.0 (85.0–182.0)	93.5 (77.0–129.0)	0.011 *
CKD-EPI GFR (mL/min/1.73 m^2^)	31.8 (27.5–64.0)	63.0 (42.2–75.8)	0.009 *
CCI (score)	6.0 (4.0–8.0)	5.0 (4.0–7.0)	0.257 *
Rockall (score)	4.0 (3.0–6.0)	4.0 (3.0–5.0)	0.394 *
Hospitalization duration (days)	8.0 (4.0–12.0)	7.0 (5.0–10.0)	0.841 *
Blood transfusion ^†^ (doses)	4.0 (2.0–6.0)	3.0 (1.0–4.0)	0.025 *
FFP transfusion (doses)	2.0 (1.2–2.7)	1.5 (0.5–2.5)	0.445 *

All data are presented as median (interquartile range). ^†^ Student’s *t*-test for independent variables, * Mann–Whitney U test.

**Table 6:** Multivariable logistic regression model showing independent predictors for the excess C_trough_ (N = 90).

Variable	OR	95% CI	*p*
Age	1.177	0.999 to 1.387	0.049
Female gender *	0.883	0.154 to 4.956	0.888
Creatinine	0.992	0.977 to 1.007	0.332
CKD-EPI GFR	0.909	0.841 to 0.982	0.016
Total bilirubin	1.016	0.927 to 1.113	0.727
Albumin	0.993	0.869 to 1.135	0.924
CCI	0.894	0.520 to 1.537	0.686
Time since the last therapy	1.046	0.955 to 1.146	0.328
Rockall score	0.592	0.326 to 1.075	0.085

OR—multivariable adjusted odds ratio; 95% CI—95% confidence interval. * Male gender is the reference group.

**Table 7:** Multiple linear regression model of independent predictors for the hospitalization duration in the study sample (N = 90).

Variable	β *	SE ^†^	t Value	*p*
Age (years)	−0.054	0.070	−0.778	0.438
RBC (×10^9^/L)	0.968	1.066	0.908	0.367
WBC (×10^9^/L)	0.203	0.088	2.296	0.024
Hb (g/L)	−0.041	0.033	−1.220	0.226
Platelets (×10^9^/L)	−0.003	0.004	−0.834	0.407
CKD-EPI GFR	0.025	0.021	1.182	0.241
PT (ratio)	1.988	2.529	0.786	0.434
APTT (sec)	0.058	0.030	1.917	0.049
Rockall (score)	0.621	0.343	−1.807	0.074
CCI (score)	0.726	0.278	2.606	0.011

* unstandardized coefficient β; ^†^ standard error.

## Data Availability

The data presented in this study are available on request from the corresponding author. The data are not publicly available due to ethical considerations.
